# Nanophotonic-Enhanced Thermal Circular Dichroism for Chiral Sensing

**DOI:** 10.1021/acsphotonics.4c01339

**Published:** 2024-12-11

**Authors:** Ershad Mohammadi, Giulia Tagliabue

**Affiliations:** † Laboratory of Nanoscience for Energy Technologies (LNET), Faculty of Engineering (STI), Ecole Polytechnique Federale de Lausanne (EPFL), Lausanne 1015, Switzerland; ‡ Department of Information in Matter and Center for Nanophotonics, AMOLF, Science Park 104, Amsterdam 1098 XG, Netherlands

**Keywords:** chiral sensing, thermal circular dichroism, thermonanophotonics, optical chirality, high-index dielectric metasurfaces, lattice resonances

## Abstract

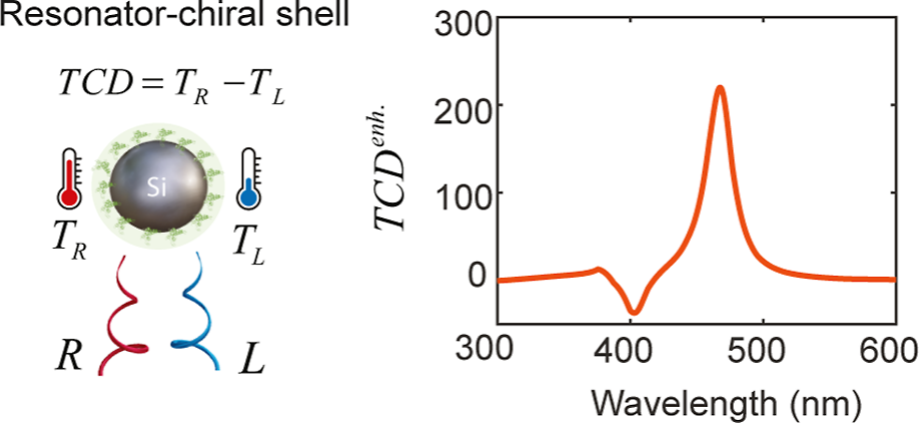

Circular dichroism (CD) can distinguish the handedness of the chiral molecules. However, it is typically very weak due to vanishing absorption at low molecular concentrations. Here, we suggest thermal CD (TCD) for chiral detection, leveraging the temperature difference in the chiral sample when subjected to right- and left-circularly polarized excitations. The TCD combines the enantiospecificity of CD with the higher sensitivity of thermal measurements while introducing new opportunities in the thermal domain that can be synergistically combined with optical approaches. We propose a theoretical framework to understand the TCD of individual and arrays of resonators covered by chiral molecules. To enhance the weak TCD of chiral samples, we first used individual dielectric Mie resonators and identified chirality transfer and self-heating as the underlying mechanisms giving rise to the differential temperature. However, inherent limitations imposed by the materials and geometries of such resonators make it challenging to surpass a certain level in enhancements. To overcome this, we suggest nonlocal thermal and electromagnetic interactions in the arrays. We predict that a combination of chirality transfer to Mie resonators, collective thermal effects, and optical lattice resonance could, in principle, offer more than four orders of magnitude enhancement in TCD. Our thermonanophotonic-based approach thus establishes key concepts for ultrasensitive chiral detection.

## Introduction

Chiral molecules exist in right- and left-handed configurations, each with distinct properties. Chiral sensing is the differentiation between these two forms, which is crucial for gaining insight into molecular structure, drug development, and monitoring environmental pollutants, among many applications.^1−3^ Circular dichroism (CD) spectroscopy can discern this handedness by measuring the contrast in the absorption of a chiral sample for right- and left-circularly polarized light. The CD signal for a sample solution is quantified as CD = 32.98Δϵ*Cl* (Supporting Information, Section S1), where Δϵ is the differential molar attenuation of the chiral species inside the sample in M^–1^ cm^–1^, *C* is the molar concentration in mol/L (usually denoted by M), and *l* is the path length in cm.^4,5^ Given a typical value of Δϵ = 20 M^–1^ cm^–1^ at molecular resonances of biomolecules in ultraviolet wavelengths,^6^ we need high concentrations and centimeter-scale path lengths to get CD signals on the order of a few millidegrees (mdeg) being within the sensitivity level of the CD spectrometers.

Optical nanoresonators have been widely used to boost weak CD signals.^7−19^ However, nanophotonic-enhanced chiral sensing schemes operating at visible wavelengths pose two challenges. First, the inherent chirality of matter diminishes significantly in the visible spectrum, nearly by two orders of magnitude when compared to the ultraviolet range.^14^ Additionally, the chiral layer interacting with the resonators has a thin profile, resulting in a very short path length (e.g., 10–50 nm). These two drawbacks should be compensated by using a higher concentration or higher enhancement factor due to the nanostructure. For instance, to get a CD value of 1 mdeg in the visible range with a path length *l* = 50 nm, we need high molar concentration *C* = 1.6 mM and an enhancement factor ∼20,000 due to the nanostructure, the latter being very challenging to reach.

One approach for nanophotonic-enhanced chiral sensing is based on exposing the chiral samples to superchiral near fields generated by the resonators.^20^ In this method, the CD enhancement is proportional to the average value of the optical chirality over the volume of the chiral sample. Yet, achieving a high-average optical chirality is challenging due to its nonuniform spatial distribution.^9,10^ Therefore, the optical chirality-based enhancements are typically less than two orders of magnitude. The second mechanism relies on the reverse interaction of chiral molecules toward resonators known as chirality transfer.^5^ The chirality transfer to achiral dielectric nanostructures can generate a much stronger CD enhancement than optical chirality-based schemes. Nevertheless, achieving enhancement levels beyond three orders of magnitude remains challenging.

Here, we exploit thermal CD (TCD) for chiral detection, which is defined as TCD = *T*_R_ – *T*_L_, where *T*_R_ and *T*_L_ are the steady-state temperatures of the chiral sample under right- and left-circularly polarized excitations, respectively. TCD can be viewed as a two-step process: the optical component arises from the different absorbed power in chiral samples for right- and left-circularly polarized excitations, and the thermal component involves the conversion of this differential absorption into a differential temperature. Such thermo-chiroptical effects have been used to identify the handedness of the chiral nanoparticles.^21−25^ Yet, applying these techniques to the chiral samples remains challenging as the cross-polarizability of chiral molecules is much weaker than that of nanoparticles. As we will show later, the TCD for chiral samples alone is very weak. However, we demonstrate that thermonanophotonics can significantly enhance it by leveraging opportunities in both the optical and thermal domains.

To gain insight into the different factors contributing to the nanophotonic-enhanced TCD, we begin by deriving closed-form formulas for the TCD of an achiral dielectric Mie resonator covered by a thin chiral shell. In this setup, we identify optical chirality transfer as the dominant mechanism giving rise to differential absorption inside resonators.^5^ Subsequently, we translate such differential absorption to the differential temperature, giving rise to the TCD of the system. Next, to further increase the TCD enhancements compared with individual resonators, we propose arrays of resonators that are electromagnetically uncoupled but thermally coupled. Here, the differential absorption is taken from the solution to the individual resonators, while the thermal aspect involves solving the conduction heat equation for an ensemble of point heaters. Finally, we add electromagnetic couplings to further amplify the enhancement factors by using collective optical modes referred to as lattice resonances (LRs).^26^ To model such coupling analytically, we employ the coupled dipole approximation (CDA) method,^27^ specifically tailored for arrays of resonators coated with chiral shells. Our proposed method computes the differential absorption due to the chirality transfer inside each resonator within the array. Afterward, we solve the conduction equation to find the steady-state temperature profile across the array.

Our findings provide key insights into the interplay between optical and thermal dynamics within systems involving chiral molecules and nanostructures. Our developed analytical methodologies allow analyzing finite chains of nanospheres covered by chiral shells, which is very time-consuming to solve with numerical simulations due to their large size compared to wavelength. We validate our analytical approaches by performing Multiphysics (optical and heat) COMSOL simulations for small arrays.

## TCD for a Mie Resonator Covered by a Chiral Shell

**Figure 1:**
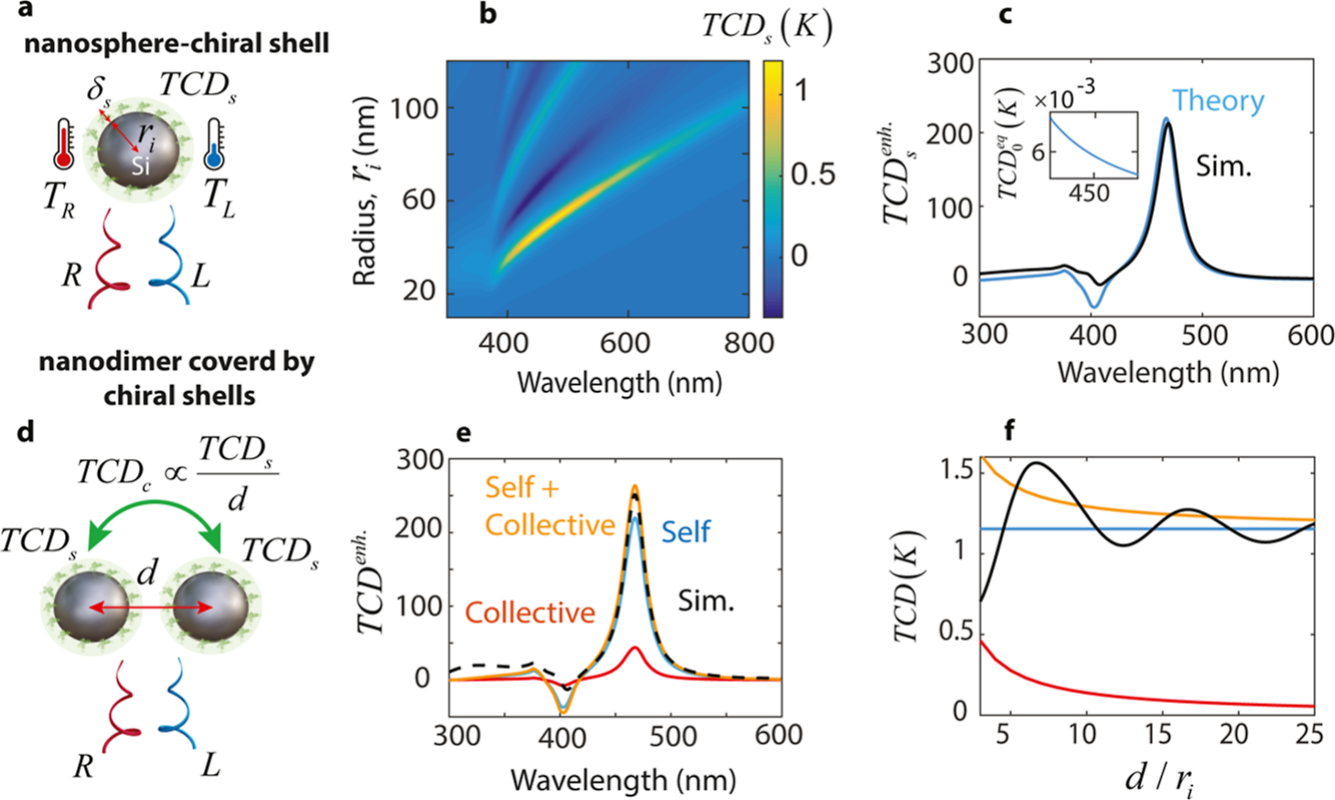
Self- and collective-TCD in dielectric nanoresonators. (a) Individual silicon nanosphere of radius *r*_*i*_ covered by a chiral shell of thickness δ_s_. The system is illuminated by right (R)- and left (L)-circularly polarized light of equal amplitudes, leading to different temperatures *T*_R_ and *T*_L_ for the sphere-shell. (b) Self-TCD of the sphere-shell (eq 1) as a function of wavelength and the radius of the nanosphere. (c) Analytical (blue) and numerical (black) estimation of the self-TCD of the sphere-shell for *r*_*i*_ = 50 nm. The inset shows the TCD of a chiral sphere with the same volume as the chiral shell. (d) Self- and collective-TCD in a dimer composed of two nanospheres placed at a distance *d* (center-to-center) and coated by chiral shells. Each sphere-shell exhibits the self-TCD similar to individual resonators. There is an additional collective-TCD from the adjacent sphere-shell due to thermal conduction. (e) Different contributions to the TCD enhancement for one of the sphere-shells in the dimer. The blue, red, and orange curves represent the self-, collective-, and total-TCD enhancements, respectively. The black curve is obtained from numerical simulations. (f) Self- (blue), collective- (red), and total- (orange) TCD as a function of the distance between nanospheres in the dimer. The wavelength is fixed at λ = 470 nm. The black curve represents the actual TCD taken from simulations.

To investigate how optical resonators can affect TCD, we first consider an achiral Mie resonator of radius *r*_*i*_ covered by a chiral shell of thickness δ_*s*_ (Figure 1a). The system is exposed to equally intense right (R)- and left (L)-circularly polarized plane waves propagating along the *z*-axis, described as , where *k*_0_ is the wavenumber. Throughout this paper, we adopt the time-harmonic convention *e*^*i*ω*t*^. The constitutive equations for a chiral medium are  and ,^28^ where κ is the Pasteur parameter accounting for the coupling between the induced electric and magnetic dipoles. We are interested in the differential temperature of the sphere-shell (i.e., TCD), which is related to the differential absorption as TCD = Δ*P*_abs_/4π*K*_0_*r*_o_, where *K*_0_ is the thermal conductivity of the surrounding medium, and *r*_o_ = *r*_*i*_ + δ_s_ is the outer radius of the thermal source (i.e., sphere-shell). The total differential absorption Δ*P*_abs_ can be decomposed into two parts: the differential absorption of the chiral shell and that of the achiral resonator. The former is due to the optical chirality produced by the resonator at the position of the chiral shell. The latter, being also the dominant one, is due to chirality transfer from the chiral shell to the achiral resonator. Here, we assume that the total differential absorption equals the chirality transfer part.^5^ In this case, we derive the TCD as (Supporting Information, Section S2)
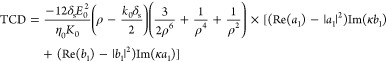
1where ρ = *k*_0_*r*, *r* = *r*_*i*_ + δ_s_/2, η_0_ is the impedance of free space, and *a*_1_ and *b*_1_ are the electric and magnetic dipolar Mie coefficients of the nanoparticle.^29^ The subscript “s” denotes the self-TCD indicating that the heat is generated by sphere-shell alone, without any influence from the collective heating effects.^30^ We obtained the TCD_s_ using eq 1 as a function of radius *r*_*i*_ and illumination wavelength (Figure 1b). The Mie resonator is a silicon sphere with realistic dispersion,^31^ which is covered by a chiral shell of thickness δ_s_ = 10 nm. The system is suspended in air with thermal conductivity *K*_0_ = 0.0262 W/m K. The Pasteur parameter and the relative permittivity of the chiral shell are κ = (1 – 0.01*i*) × 10^–2^ and ε_r_ = 1.33^2^ – 0.001*i*, respectively. We should note that the realistic value of the Pasteur parameter may be significantly lower (Supporting Information and Section S1), but we use this value to ensure the numerical accuracy of simulations within a reasonable computational time. The incident power is 50 MW/m^2^, which corresponds to an incident electric field *E*_0_ = 1.3725 × 10^5^ V/m. The spectra in Figure 1b show a maximized TCD of 1.2 K for radius *r*_*i*_ = 50 nm and λ = 470 nm, which is due to the maximum chirality transfer from the chiral shell to the magnetic dipolar resonance of the silicon nanosphere.^5^ To get the enhancement value, we normalize the TCD to that of an equivalent chiral sphere (TCD_0_^eq^), which has the same permittivity, Pasteur parameter, and volume as the chiral shell (Supporting Information, Section S3). By applying this normalization, we obtain the TCD enhancement for *r*_*i*_ = 50 nm (Figure 1c, blue curve). The inset represents the TCD_0_^eq^ reaching 0.0053 K at λ = 470 nm. Notably, the Mie resonator amplifies this TCD by a factor of 225. To check the validity of our derivation in eq 1, we compute the same enhancement from numerical simulations (Figure 1c, black curve), where we perform Multiphysics thermal and optical simulations. We see that the theory and simulations are in very good agreement. A crucial aspect of our thermonanophotonic chiral detection scheme is to ensure that the maximum temperature of the chiral molecules remains below the tolerance limit. At the same time, the TCD should be above the sensitivity level of the differential thermometry measurements.^32,33^ The maximum temperature is the sum of the initial temperature and the temperature increase, where the latter depends linearly on the incident power. The TCD is influenced by both the incident power and the Pasteur parameter. By selecting appropriate values for these parameters, we can keep the maximum temperature within safe limits while ensuring a detectable TCD (Supporting Information, Section S4).

## TCD for a Nanodimer Covered by Chiral Shells

To obtain the enhancement beyond that achieved by single resonators, we exploit collective effects in multiple resonators. In the simplest case, we examine a nanodimer composed of two silicon spheres of radius *r*_*i*_ = 50 nm, positioned at center-to-center distance *d*, and both covered by chiral shells of thickness δ_s_ = 10 nm (Figure 1d). In this configuration, in addition to the self-TCD (TCD_s_), there is a collective contribution (TCD_c_) to the total-TCD of each sphere-shell. This component arises from thermal conduction, which conveys the self-TCD of each sphere-shell to the neighboring particle through a factor of 1/4π*K*_0_*d*. We plot the self- and collective-TCD enhancements for *d* = 5*r*_*i*_ in Figure 1e (blue and red curves, respectively). The self-part is the same as the blue curve in Figure 1c, while the collective component is TCD_c_ = TCD_s_/4π*K*_0_*d* normalized to TCD_0_^eq^. The total enhancement (orange curve) is (TCD_s_ + TCD_c_)/TCD_0_^eq^, which is aligned with the actual value taken from numerical simulations (black curve). We see that by introducing an additional particle, the TCD enhancement reaches 270, indicating a 1.2-fold increase compared to an individual sphere-shell.

In the dimer shown in Figure 1d, we overlooked electromagnetic coupling between sphere-shells. To investigate this coupling effect, we plot the self- (blue), collective- (red), and total- (orange) TCD as a function of separation distance *d* (Figure 1f). We see that the total-TCD can estimate the actual TCD. Yet, adding electromagnetic coupling, especially at short distances (*d* < 6*r*_*i*_), is crucial. For large separations, the total and actual TCDs converge due to weak electromagnetic interactions between sphere-shells. Furthermore, the total and actual TCDs are very close at *d* = 5*r*_*i*_, explaining the good agreement between the orange and black curves in Figure 1e.

## Resonator Arrays: Thermally Coupled and Electromagnetically Uncoupled

**Figure 2:**
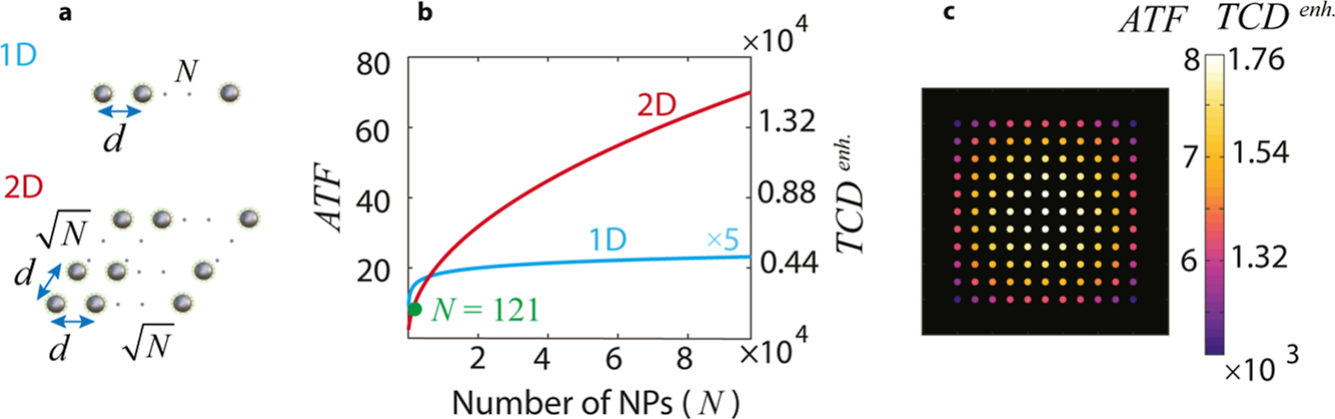
Harnessing the collective TCD in 1D and 2D arrays of electromagnetically uncoupled resonators. (a) 1D and 2D arrays made of *N* nanosphere-shells with distance *d* between them. (b) Array thermal factor (left-axis) and TCD enhancement (right-axis) for the central sphere-shell as a function of number of nanoparticles (*N*). Blue and red curves correspond to 1D and 2D arrays. (c) ATF and TCD^*enh*^ across the entire array for a 2D array made of *N* = 121 (11 × 11) sphere-shells. For the TCD^*enh*^ calculations in (b,c), the TCD_s_ and TCD_0_^eq^ are that of the individual resonator studied in Figure 1c, the separation distance is *d* = 5*r*_*i*_, and the wavelength is λ = 470 nm.

The enhancement in TCD obtained through the thermal coupling, and enabled by the simple and long-range 1/*d* factor, suggests that adding more resonator can further boost this effect.^34^ If we consider an array of *N* nanoresonators each coated by a chiral shell (Figure 2a), then in the uncoupled electromagnetic regime, the collective-TCD for the *n*-th sphere-shell is expressed as
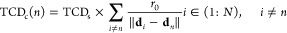
2where **d**_*i*_ is the position vector to the *i*-th sphere-shell and TCD_s_ is the self-TCD of an individual sphere-shell. Therefore, the total-TCD for the *n*-th sphere-shell is given by TCD(*n*) = TCD_s_ + TCD_c_(*n*), and the corresponding enhancement is expressed as
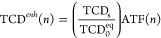
3where ATF is the array thermal factor, indicating the TCD enhancement factor due to the thermal interactions within the array, and defined as
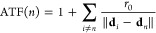
4Equation 3 breaks down the TCD enhancement into two components: the chirality transfer from the chiral shell to the underlying resonator (TCD_s_/TCD_0_^eq^) and the collective thermal effects (ATF). As these two components are multiplied together, even a small increase in one can result in a substantial change in the total TCD enhancement. To demonstrate this, we consider arrays of sphere-shells with the same parameters as in Figure 1 and a separation distance *d* = 5*r*_*i*_. We depict the ATF for the central sphere-shell in one-dimensional (1D) (blue curve) and two-dimensional (2D) (red curve) arrangements as a function of the number of nanoparticles *N* (left axis in Figure 2b). We see that by increasing the number of sphere-shells to 10^4^, the ATF for 1D and 2D arrays can reach values of up to 4.6 and 70, respectively. Considering an operating wavelength of λ = 470 nm, multiplying this ATF by the TCD enhancement of an individual sphere-shell (i.e., 220) yields the corresponding TCD enhancements within the array as 1018 and 1.54 × 10^4^, respectively (right axis in Figure 2b). To investigate the position dependency in the array, we depict the ATF and TCD^*enh*^ across a 2D array composed of 121 nanoparticles arranged in an 11 × 11 lattice with a spacing *d* = 5*r*_*i*_ (Figure 2c). We see that the maximum enhancement occurs at the center of the array, which is always the case when we have no electromagnetic coupling between resonators. Furthermore, in the center of the array, ATF ≈ 8 and TCD^*enh*^ ≈ 1756, corresponding to the green dot in Figure 2b.

## Resonator Arrays: Thermally and Electromagnetically Coupled

The analytical prediction of TCD in the uncoupled electromagnetic regime starts deviating from numerical simulation for *d* < 6*r*_*i*_ (Figure 1f). On the other hand, 1/*d* decay of the collective-TCD suggests keeping separation distances small to effectively capture thermal interactions. This underscores the importance of considering electromagnetic couplings to make more accurate analytical approximations for shorter separation distances.

To accomplish this, we must characterize the differential absorption of each sphere-shell within the array. This serves as the source term for the thermal conduction equation, enabling us to compute the self-, collective-, and total-TCD of each sphere-shell. We begin our approach by applying the CDA method to the array of resonators without the chiral shells (Supporting Information, Section S5). In the CDA method, we replace each sphere by an electric and a magnetic dipole and then the electromagnetic interaction between these dipoles is computed through solving a self-consistent system of equations. The output of this system is the local fields (***E***_loc,R/L_^w.o^, ***H***_loc,R/L_^w.o^) at the position of each sphere and the corresponding induced dipole moments (***p***_R/L_^w.o^, ***m***_R/L_^w.o^). The superscript “w.o” denotes that the quantities are associated with the sphere array alone and excluding the chiral shells. As the sphere array alone is achiral, such local fields and dipole moments have the same amplitudes for right- and left-circular polarizations (i.e., |***E***_loc,R_^w.o^| = |***E***_loc,L_^w.o^|, |***H***_loc,R_^w.o^| = |***H***_loc,L_^w.o^|, |***p***_R_^w.o^| = |***p***_L_^w.o^|, |***m***_R_^w.o^| = |***m***_L_^w.o^|). Using the free-space Green’s functions and the induced dipole moments ***p***_R/L_^w.o^ and ***m***_R/L_^w.o^, we can calculate the near field around each resonator (***E***_s,R/L_ and ***H***_s,R/L_) and at the position of the surrounding chiral shell. Next, we cover the resonators with chiral shells and assume that the presence of the shell does not disturb ***E***_s,R/L_ and ***H***_s,R/L_. This assumption is correct as far as the chiral shells are thin and their refractive index is close to the surrounding medium. Given this assumption, the electric (***P***_s,R/L_) and magnetic (***M***_s,R/L_) polarization densities over the chiral shells are related to the near fields as ***P***_s,R/L_ = ϵ_0_(ϵ_r_ – 1)***E***_s,R/L_ – *i*κ***H***_s,R/L_/*c*_0_ and ***M***_s,R/L_ = *i*κ***E***_s,R/L_/η_0_, respectively. To calculate the chirality transfer, we need to find the back-action of such polarization on the underlying resonator. This results in the new local fields and the dipole moments for right- and left-circular excitations being different now due to the presence of chirality in the system (i.e., |***E***_loc,R_^w^| ≠ |***E***_loc,L_^w^|, |***H***_loc,R_^w^| ≠ |***H***_loc,L_^w^|, |***p***_R_^w^| ≠ |***p***_L_^w^|, |***m***_R_^w^| ≠ |***m***_L_^w^|). The superscript “w” indicates that the quantities correspond to the sphere array coated with chiral shells. We can obtain the extinct and scattered powers by each sphere-shell as
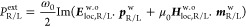
5
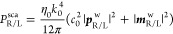
6where ω_0_ is the angular frequency. Lastly, the differential extinct, scattered, and absorbed powers due to chirality transfer can be expressed as Δ*P*^ext^ = *P*_R_^ext^ – *P*_L_^ext^, Δ*P*^sca^ = *P*_R_^sca^ – *P*_L_^sca^, and Δ*P*^abs^ = Δ*P*^ext^ – Δ*P*^sca^, where the latter is of particular interest for our problem as it determines the self-TCD of the *n*’th sphere-shell as
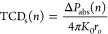
7

Then, the total TCD for the *n*-th sphere-shell reads as
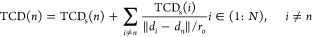
8

**Figure 3:**
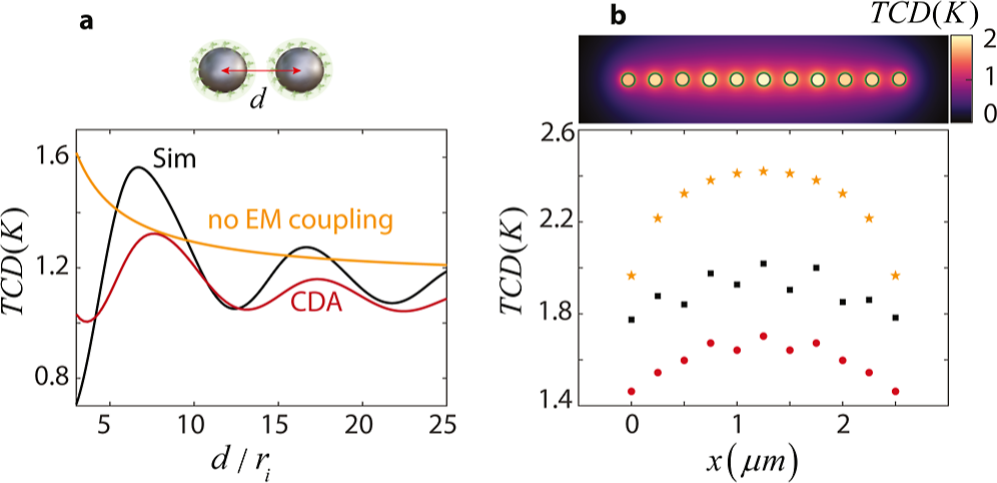
Effect of electromagnetic coupling on the TCD of sphere-shell arrays. (a) TCD of one of the sphere-shells for the dimer in Figure 1d as a function of separation distance *d*. The orange curve corresponds to the case with no electromagnetic coupling (same as the orange curve in Figure 1f). The red curve is obtained from CDA, and the black curve is actual TCD based on numerical simulations (same as the black curve in Figure 1f). (b) TCD across a 1D array made of *N* = 11 sphere-shells separated by distance *d* = 5*r*_*i*_. The parameters of the system are the same as those in Figure 1) and the operating wavelength is λ = 470 nm. The top panel shows the simulated TCD. In the bottom panel, the orange stars indicate TCD in the uncoupled electromagnetic regime, the red circles correspond to the TCD obtained from CDA, and the black rectangles denote the TCD obtained from numerical simulations.

To check the validity of the proposed method, we examine the same dimer in Figure 1d and obtain the TCD of one of the nanoparticles as a function of separation distance *d* (red curve in Figure 3a). Comparing the result from CDA with that obtained based on no electromagnetic coupling (orange curve), we observe that the former aligns more closely with the actual TCD (black curve), thereby offering more accurate predictions, particularly for small separation distances. Next, we investigate the TCD profile across a 1D array made of *N* = 11 sphere-shells placed at a distance *d* = 5*r*_*i*_ (top panel in Figure 3b). The parameters for spheres and shells are the same as those in previous sections, and the wavelength is λ = 470 nm. Again, we obtain the TCD using the CDA method (red circles in bottom panel in Figure 3b) and compare it to the case when there is no electromagnetic coupling in the array (orange stars) as well as to the actual TCD taken from numerical simulations (black rectangles). Notably, we see that the CDA method not only aligns closer to the simulation but also captures the real temperature profile.

We note that there is yet a discrepancy between CDA-based approximations and numerical simulations. This error could be attributed to the differential absorption inside the chiral shells due to the optical chirality, which is neglected because of the dominance of chirality-transfer-based differential absorption. Furthermore, the contrast in the refractive index of the chiral sample (1.33^2^ – 0.001*i*) and the surrounding medium (in this case, air) contributes to the error. This alters the actual polarizing near fields acting on the chiral shells compared to those of the array without chiral shells (i.e., ***E***_s,R/L_ and ***H***_s,R/L_).

## Combining Collective Thermal Interactions and Optical Lattice Resonances

**Figure 4:**
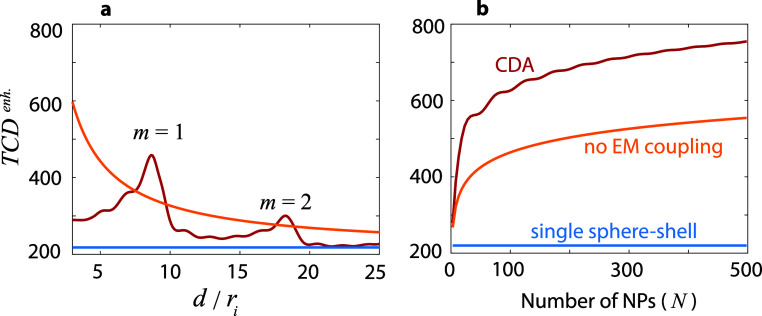
Synergy of collective thermal interactions and optical LRs in a 1D array of sphere-shells. The TCD enhancement in the central sphere-shell (a) as a function of separation distance *d* for *N* = 11 nanoparticles and (b) as a function of number of nanoparticles (*N*) for *d* = 8.6*r*_*i*_. In both (a,b), the red curve corresponds to the CDA-based calculation. The orange curve shows enhancement without electromagnetic coupling. The blue curve denotes the base-enhancement of an individual sphere-shell.

In Figure 3b, we noted that the TCD achieved by the 1D array made of *N* = 11 sphere-shells with a spacing *d* = 5*r*_*i*_ reaches 1.7, corresponding to a 320-fold enhancement. To further boost electromagnetic interactions, we propose LRs emerging from the radiative coupling between nanoparticles through in-plane diffraction.^35^ For an infinite array, the *m*-th order of such collective Mie modes occurs at spacing *d* = *m*λ. To verify this, we calculate the TCD enhancement using CDA as a function of spacing in the central sphere-shell of the array examined in Figure 3b (red curve in Figure 4a). The first and second order LRs are evident for *d*(*m* = 1) = 8.6*r*_*i*_ and *d*(*m* = 2) = 18.25*r*_*i*_, corresponding to *d* = 0.91λ and *d* = 1.94λ, as expected. For comparison, we also depict the TCD enhancement in the uncoupled electromagnetic regime (orange curve) as well as the baseline enhancement provided by an individual resonator (blue curve). We see that the LRs can increase the TCD beyond the uncoupled regime, reaching 500-fold enhancement at the strongest order (i.e., *m* = 1). Next, we investigate the effect of the number of nanoparticles (*N*) on the TCD for a fixed spacing *d*(*m* = 1) = 8.6*r*_*i*_ (Figure 4b). We see that by increasing *N* higher enhancement factors up to 700-fold are possible. We anticipate that for the 2D arrays of sphere-shells, LRs could achieve TCD enhancement factors even greater than the corresponding noncoupled regime (see the red curve in Figure 2b). We should note that when the array is embedded in a medium with a refractive index greater than one or placed on a substrate, the LRs may weaken in strength or broaden spectrally.^26^

## Chiral Sensitivity of TCD

To explore the potential of TCD for ultrasensitive chiral detection, we compare its sensitivity to that of the CD by investigating two analytically tractable systems (Supporting Information Section S6). The first is a chiral slab of thickness *l* = 5 nm and Pasteur parameter Im(κ) = 10^–6^, considered for CD studies. The second system is a 15 μm square array consisting of 1000 × 1000 chiral spheres of radius *r* = 10 nm and spacing *d* = 15 nm, intended for TCD analysis. Both systems are in free space and illuminated by right- and left-circularly polarized light of incident power 50 MW/m^2^. Notably, the amount of chiral material in the chiral slab is greater than in the sphere array as the chiral slab is continuous, whereas the array is finite and contains empty space between the spheres. The CD of the chiral slab, expressed in degrees, is given by *k*_0_ Im(κ)*l*(180/π) (Supporting Information Section S1), which results in a CD of ≈0.008 mdeg at wavelength λ = 470 nm. Even with high CD sensitivities, such as 1 mdeg, this value remains more than two orders of magnitude below the detection limit. Next, we quantify the TCD for the sphere array. The TCD for a small chiral sphere is expressed as (Supporting Information Section S3)
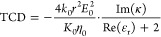
9Substituting the values as in the previous sections into this formula yields TCD = 6.8 × 10^–4^ mK. However, within the array, the ATF for the central sphere reaches 1150, thereby increasing the TCD to ≈0.8 mK. Millikelvin accuracy in temperature measurement has already been demonstrated using advanced nanothermometry techniques such as probing of single-atomic defects in diamond.^36,37^ Moreover, there is a variety of techniques such as optical interferometry and thermoreflectance being able to address the required temperature measurement resolutions along with nanometric spatial resolutions.^33^

In conclusion, we suggested TCD combined with nanophotonics for the ultrasensitive detection of molecular chirality. We demonstrated our thermo-nanophotonic approach analytically and numerically for very thin chiral coatings on spherical silicon resonators. We identified the chirality transfer as the main underlying source of TCD, and based on this, we derived closed-form formulas for the TCD of an individual resonator-shell. Furthermore, in our quest toward higher enhancement factors, we proposed arrays, where the thermal and optical properties of the problem can be synergistically combined. We showed that the collective thermal effects in combination with the optical LR can increase the TCD substantially beyond the individual resonator level. Our findings open new avenues for advancement in ultrasensitive chiral detection.

## Supporting Information Available

Obtaining the CD of a chiral slab using constitutive equations and the Beer–Lambert law; TCD of an achiral resonator covered by a chiral shell; TCD of a small chiral sphere; temperature analysis; chirality transfer in an array of nanoresonators covered by chiral shells; and comparison between the chiral sensitivity of CD and TCD (PDF)
