# Supplementary material for: Nanophotonic-Enhanced Thermal Circular Dichroism for Chiral Sensing
Source: ACS Photonics. 2024 Dec 11;12(1):152–8. doi: 10.1021/acsphotonics.4c01339 (PMC11741133; doi:10.1021/acsphotonics.4c01339)
Supplement: Supplementary file 1 — ph4c01339_si_001.pdf [file ph4c01339_si_001.pdf]

Supporting Information for

Nanophotonic-Enhanced Thermal Circular Dichroism for Chiral Sensing

Ershad Mohammadi[1,2], Giulia Tagliabue[1*]

[1] Laboratory of Nanoscience for Energy Technologies (LNET), École Polytechnique Fédérale de Lausanne (EPFL), Lausanne 1015, Switzerland

[2] Department of Information in Matter and Center for Nanophotonics, AMOLF, Science Park 104, 1098 XG Amsterdam, Netherlands.

*Email: giulia.tagliabue@epfl.ch

Contents:

Supporting Section S1. Obtaining the circular dichroism of a chiral slab using: (A) constitutive equations, and (B) Beer–Lambert law

Supporting Section S2. The Thermal Circular Dichroism (TCD) of an achiral resonator covered by a chiral shell

Supporting Section S3. Thermal circular dichroism of a small chiral sphere

Supporting Section S4. Temperature analysis

Supporting Section S5. Chirality transfer in an array of nanoresonators covered by chiral shells

Supporting Section S6. Comparison between the chiral sensitivity of CD and TCD

Supporting Section S1. Obtaining the circular dichroism of a chiral slab using: (A) constitutive equations, and (B) Beer–Lambert law

S1-A. We consider a chiral slab of thickness l which is illuminated by the right (“*R*”)- and the left (“*L*”)-circularly polarized light propagating along the *z*-axis. (Figure S1). The electric field at the left side of the

slab ($z = 0$) is defined as $\boldsymbol{E}_{R/L}\left(z=0\right) = E_0 \exp(-ik_0 z)\, \hat{e}_{R/L}$, where k_0 is the wavenumber in free space. $\hat{e}_R = \left(\hat{x} - i\hat{y}\right)$ and $\hat{e}_L = \left(\hat{x} + i\hat{y}\right)$ are the base vectors for the right- and the left-circular polarizations, respectively. The chiral slab has the refractive index n and the Pasteur parameter κ. Thus, the effective refractive indices for right- and left-circular polarizations propagating through the slab, are $n_R = n + \kappa$ and $n_L = n - \kappa$, respectively. This renders the corresponding fields at the output side as:

$$\boldsymbol{E}_{R/L}\left(z = l\right) = E_0 \exp(-ik_0 n_{R/L} l)\, \hat{e}_{R/L} \qquad \text{(S1)}$$

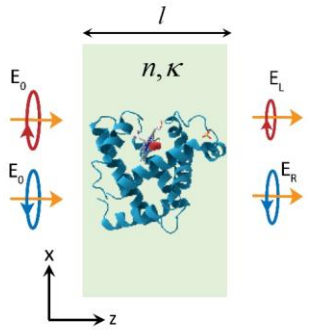

Figure S1. A chiral slab of thickness l which is illuminated by the right- and the left-circularly polarized light of equal amplitudes E_0. n and κ represent the refractive index and the Pasteur parameter of the chiral substance.

If E_R and E_L are the magnitudes of the electric field vectors for the right- and left-circular polarizations at the output side, then the CD is reported as $CD = \tan^{-1}\left[\left(E_R - E_L\right)/\left(E_R + E_L\right)\right]$, which by assuming small ellipticity ($\tan\theta \approx \theta$) the CD signal is expressed as:

$$CD \approx \left(\frac{E_R - E_L}{E_R + E_L}\right) \qquad \text{(S2)}$$

By putting the electric field amplitudes from Equation S1 in Equation S2:

$$\frac{E_R - E_L}{E_R + E_L} = \frac{\exp\left(k_0\left[\mathrm{Im}(n) + \mathrm{Im}(\kappa)\right]l\right) - \exp\left(k_0\left[\mathrm{Im}(n) - \mathrm{Im}(\kappa)\right]l\right)}{\exp\left(k_0\left[\mathrm{Im}(n) + \mathrm{Im}(\kappa)\right]l\right) + \exp\left(k_0\left[\mathrm{Im}(n) - \mathrm{Im}(\kappa)\right]l\right)} = \sinh\left(k_0\,\mathrm{Im}(\kappa)\,l\right) \qquad \text{(S3)}$$

If $k_0\,\mathrm{Im}(\kappa)\,l << 1$, then the CD (in degrees) can be expressed as:

$$CD\left(\deg\right) \approx k_0 \mathrm{Im}\left(\kappa\right)\, l \left(\frac{180}{\pi}\right) \qquad \text{(S4)}$$

S1-B. Next, we describe the same chiral system in Figure S1 according to the Beer–Lambert law, where the optical attenuation of light can be expressed as:

$$A_{R/L} = \log\left(I_0/I_{R/L}\right) = \varepsilon_{R/L}\ C\ l \qquad \text{(S5)}$$

where A is the absorbances of the sample, which is defined as the decadic logarithm of the ratio of incident (I_0) to the transmitted power intensity (I) as $A = \log\left(I_0/I\right)$. C is the molar concentration in mol/L (usually denoted by letter M) and l is the path length in cm. ε is the molar extinction cross section in $\mathrm{M^{-1}cm^{-1}}$ which is related to the extinction cross section of species inside the sample (σ in $\mathrm{cm^2}$) as $\varepsilon = \sigma\left(2.6157\times10^{20}\right)$. The output intensities for RCP and LCP (i.e., I_R and I_L) are connected to the corresponding absorbances (i.e., A_R and A_L) as $I_{R/L} = I_0 \times 10^{-A_{R/L}}$. Thus, the output amplitudes of E_R and E_L are related to A_R and A_L as:

$$E_{R/L} = \sqrt{I_0}\ \exp\left(-A_{R/L}\ \ln\left(10\right)/2\right) \qquad \text{(S6)}$$

Putting E_R and E_L from Equation S6 in Equation S2, gives the CD as:

$$CD\left(rad\right) \simeq \left(\frac{E_R - E_L}{E_R + E_L}\right) = \frac{\exp\left(\dfrac{-\Delta A\ \ln\left(10\right)}{2}\right) - 1}{\exp\left(\dfrac{-\Delta A\ \ln\left(10\right)}{2}\right) + 1} \qquad \text{(S7)}$$

If $\Delta A << 1$, then CD (in degrees) can be expressed in terms of the differential absorbance ($\Delta A = A_R - A_L$) as:

$$CD = \Delta A\, \frac{\ln\left(10\right)}{4}\ \frac{180}{\pi} = 32.98\ \Delta A \qquad \text{(S8)}$$

Next, combining Equation S8 and Equation S5 gives,

$$CD = 32.98\ \Delta\varepsilon\ C\ l \qquad \text{(S9)}$$

where $\Delta\varepsilon = \Delta\sigma\left(2.6157\times10^{20}\right)$ is the differential molar attenuation of the species inside the chiral sample in $\mathrm{M^{-1}cm^{-1}}$, being $\Delta\sigma$ as the differential extinction cross section of species inside the chiral solution in

cm^2. Finally, by equating Equation S4 with Equation S9, we can rephrase the imaginary part of Pasteur parameter as:

$$\mathrm{Im}(\kappa) = 0.0916\,\lambda_0\; C\;\; \Delta\varepsilon \qquad \text{(S10)}$$

where λ_0 is the wavelength in cm. As an example, a typical value of $\Delta\varepsilon = 20\ \mathrm{M^{-1}cm^{-1}}$ (at molecular resonance),[1] $C = 1.6\ \mathrm{mM}$, and a resonance wavelength $\lambda = 220\ nm$, gives the Pasteur parameter $\kappa = 6.44\times10^{-8}$ which is extremely small. However, achieving higher values of κ is possible by increasing the concentration or employing chiral substances in solid phase.[2]

Supporting Section S2. The Thermal Circular Dichroism (TCD) of an achiral resonator covered by a chiral shell

Figure S2 shows an achiral nanosphere of radius r_i covered by a thin chiral shell of thickness δ_s. The constitutive equations for the chiral medium are described as:

$$\begin{aligned} \boldsymbol{D} &= \varepsilon_0\varepsilon_r\boldsymbol{E} - i\,\frac{\kappa}{c_0}\boldsymbol{H} \\ \boldsymbol{B} &= \mu_0\boldsymbol{H} + i\,\frac{\kappa}{c_0}\boldsymbol{E} \end{aligned} \qquad \text{(S11)}$$

where ε_r is the relative permittivity and κ is the Pasteur parameter representing the chirality of the medium. The whole system is illuminated by right- and left-circularly polarized light propagating along the *z*-axis. The incident electric and magnetic fields can be expressed as:

$$\begin{aligned} \boldsymbol{E}_{R/L} &= E_0 \exp(-ik_0 z)(\hat{x} \mp i\hat{y}) \\ \boldsymbol{H}_{R/L} &= \frac{\pm iE_0}{\eta_0}\exp(-ik_0 z)(\hat{x} \mp i\hat{y}) \end{aligned} \qquad \text{(S12)}$$

where the upper (lower) sign in " $\pm\,/\mp$ " is used for the right- (left)-circularly polarized excitation, and η_0 is impedance of free space. We adopt the time-harmonic convention $\exp(i\,\omega t)$. The differential absorption in resonator-shell shown in Figure S2 is defined as the difference between the optical absorbed power for right and left-circularly polarized excitations (i.e., $\Delta P_{abs} = P_{abs,R} - P_{abs,L}$). Without the chiral shell, the differential absorption is zero as the achiral resonator has the same optical response for both polarizations. In the presence of the chiral shell, there are two sources of the differential absorption. The first one is due to the optical chirality of the near field at the position of the chiral shell. The optical chirality

is defined as $C = \frac{-k_0}{2c_0}\mathrm{Im}\left(\boldsymbol{E}\cdot\boldsymbol{H}^*\right)$, where $\boldsymbol{E}$ and $\boldsymbol{H}$ are the complex electric and magnetic fields at the molecular position, and k_0 and c_0 are the wavenumber and speed of light in free space, respectively. Optical chirality can be enhanced beyond its value for circularly polarized plane waves. The differential absorption due to the optical chirality is proportional to the average value of optical chirality produced by the resonator at the position of each chiral shell.

The second source is the transfer of the chiral response from the shell to the achiral resonator through dipole–dipole interactions. Such chirality transfer perturbs the local fields at the position of the achiral resonator, which would be otherwise equal under excitation with right- and left-circular polarizations. It has been shown that the chirality transfer dominates the total differential absorption for dielectric resonators while the contribution of optical chirality is negligible. The differential absorption due to chirality transfer can be expressed as:[3]

$$\Delta P_{abs} = \frac{-48\pi\delta_s}{\eta_0 k_0} E_0^2 \rho^2 \left(\frac{3}{2\rho^6} + \frac{1}{\rho^4} + \frac{1}{\rho^2}\right) \left[\left(\mathrm{Re}\left(a_1\right) - \left|a_1\right|^2\right)\mathrm{Im}\left(\kappa b_1\right) + \left(\mathrm{Re}\left(b_1\right) - \left|b_1\right|^2\right)\mathrm{Im}\left(\kappa a_1\right)\right] \tag{S13}$$

where $\rho = k_0 r$, and $r = r_i + \delta_s / 2$. a_1, b_1 are the dipolar coefficients of the Mie expansion of the achiral nanoparticle. The TCD of the resonator-shell is given by $TCD = \Delta P_{abs} / 4K_0 r_o$, which can be expressed as:

$$TCD = \frac{-12\delta_s E_0^2}{\eta_0 K_0}\left(\rho - \frac{k_0\delta_s}{2}\right)\left(\frac{3}{2\rho^6} + \frac{1}{\rho^4} + \frac{1}{\rho^2}\right) \left[\left(\mathrm{Re}\left(a_1\right) - \left|a_1\right|^2\right)\mathrm{Im}\left(\kappa b_1\right) + \left(\mathrm{Re}\left(b_1\right) - \left|b_1\right|^2\right)\mathrm{Im}\left(\kappa a_1\right)\right] \tag{S14}$$

where K_0 is thermal conductivity of the surrounding medium, and $r_0 = r_i + \delta_s$ is the outer radius of the thermal source (i.e., sphere-shell).

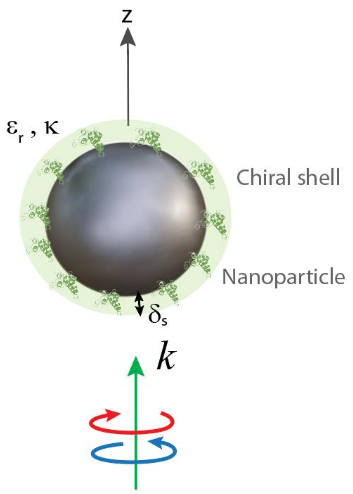

Figure S2. An achiral nanoparticle surrounded by a thin chiral shell of thickness δ_s. The system is illuminated by the right- and the left-circularly polarized plane waves of the same amplitudes.

Supporting Section S3. Thermal circular dichroism of a small chiral sphere

We consider a small chiral sphere of radius r_c which is illuminated by right- and left-circularly polarized plane waves of the equal amplitudes as:

$$\boldsymbol{E}_{inc,R/L} = E_0 \exp(-ik_0 z)\, \hat{e}_{R/L} \qquad \text{(S15)}$$

where $\hat{e}_R = (\hat{x} - i\hat{y})$ and $\hat{e}_L = (\hat{x} + i\hat{y})$ are the base vectors for the right- and the left-circular polarizations, respectively (Figure S3). The differential absorbed power for such chiral sphere can be expressed as:

$$\Delta P_0 = -\,4\omega_0 E_0^2 \,\mathrm{Re}(\alpha_{em}) / \eta_0 \qquad \text{(S16)}$$

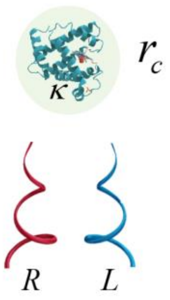

Figure S3. A small chiral sphere of radius r_c illuminated by right- and left-circularly polarized light of the same amplitude.

where α_{em} is the quasi-static cross-polarizability of the chiral sphere given as:[4]

$$\alpha_{em} = 4\pi\varepsilon_0\eta_0 r_c^3 \frac{-3i\kappa}{(\mu_r+2)(\varepsilon_r+2)-\kappa^2} \qquad \text{(S17)}$$

in which κ, ε_r, and μ_r are the Pasteur parameter, relative permittivity, and relative permeability of the chiral sphere. Putting Equation S17 in Equation S16 and assuming very small Pasteur parameter ($\kappa^2 \simeq 0$) as well as a small imaginary part of the permittivity compared to its real part ($\varepsilon_r \simeq \mathrm{Re}(\varepsilon_r)$), we can rephrase ΔP_0 as:

$$\Delta P_0 = -\frac{16\pi k_0 r_c^3 E_0^2}{\eta_0}\frac{\mathrm{Im}(\kappa)}{\mathrm{Re}(\varepsilon_r)+2} \qquad \text{(S18)}$$

Therefore, the TCD of the chiral sphere can be expressed as:

$$TCD = \frac{\Delta P_0}{4\pi K_0 r_c} = -\frac{4k_0 r_c^2 E_0^2}{K_0\eta_0}\frac{\mathrm{Im}(\kappa)}{\mathrm{Re}(\varepsilon_r)+2} \qquad \text{(S19)}$$

where K_0 is the thermal conductivity of the surrounding medium. Figure S4 shows the TCD of a chiral sphere of radius r_c computed using Multiphysics optical and heat simulations (blue) as well as by utilizing Equation S19 (orange). We see that the theory is valid for $k_0 r_c < 0.8$ (i.e., $r_c < 65\ nm$ at $\lambda = 500\ nm$). For larger chiral spheres the simulation and theory start to deviate which is due to inaccurate quasi-static approximation utilized in deriving Equations S19.

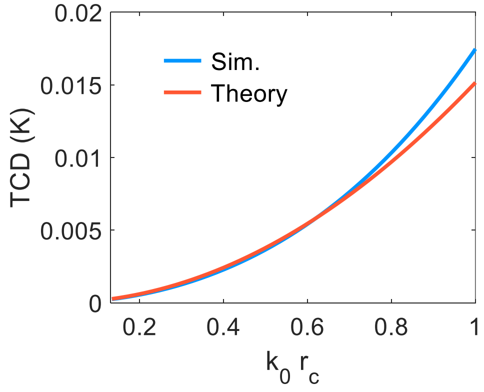

Figure S4. The thermal circular dichroism of a chiral sphere of radius r_c in free space obtained by full-wave numerical simulations (blue) and based on Equation S19 (orange). The chiral sphere is illuminated by right- and left

circularly polarized plane waves as shown in Figure S3. The illumination wavelength is $\lambda = 500\ nm$ and its power flux density is $S = \left(E_0^2 / \eta_0\right) = 5\times10^7 W / m^2$. Thermal conductivity of the surrounding medium is considered as $K_0 = 0.026\ W / m\ K$. The Pasteur parameter and the permittivity of the chiral sphere are $\kappa = (1-0.01i)\times10^{-2}$ and $\varepsilon_r = 1.33^2 - 0.001i$, respectively.

For a chiral shell of thickens δ_s surrounding a spherical nanoparticle of radius r_i , the radius of equivalent sphere (a sphere with the same volume of the chiral shell) is $r_{eq} = \sqrt[3]{3r^2\delta_s}$, where $r = r_i + \delta_s / 2$. By putting this radius in Equation S19, the TCD of equivalent sphere can be obtained as:

$$TCD = \frac{-12k_0\delta_s^{2/3}r^{4/3}E_0^2}{\sqrt[3]{3}\eta_0 K_0}\frac{\mathrm{Im}\left(\kappa\right)}{\mathrm{Re}\left(\varepsilon_r\right)+2} \qquad \text{(S20)}$$

From Figure S4 we see that the theoretical prediction of the TCD of a chiral sphere is accurate for $k_0 r_c < 0.8$ which means $r_c < 65\ nm$ at $\lambda = 500\ nm$. Recalling $r_{eq} = \sqrt[3]{3r^2\delta_s}$ relation, this indicates that the TCD of the equivalent sphere of the chiral shell obtained from Equation S19, is valid for $r_i < 90\ nm$.

Supporting Section S4. Temperature analysis

A crucial aspect of our thermonanophotonic chiral detection scheme is to ensure that the maximum temperature of the chiral molecules remains below their tolerance limit. At the same time the TCD should be above the sensitivity level of the differential thermometry measurements. The maximum temperature is the sum of the initial temperature and the temperature rise. Here, we examine the temperature rise for individual and arrays of resonator-shells and propose mechanisms to maintain the maximum temperature within a specified limit while keeping TCD high enough.

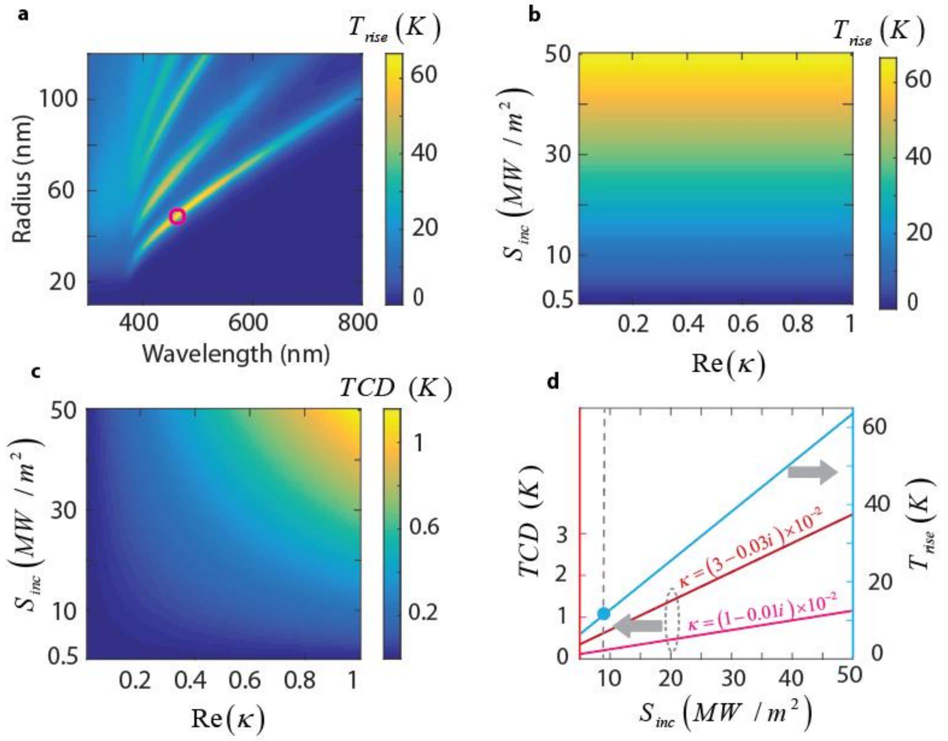

Figure S5. The temperature analysis for a silicon nanosphere covered by a thin chiral shell. (a) The temperature rise as a function of wavelength and radius of the nanosphere. The incident power flux density is $S_{inc} = 50\ MW\ /\ m^2$ and the Pasteur parameter of the chiral shell is $\kappa = (1-0.01i)\times10^{-2}$. (b) The temperature rise and, (c) the TCD as a function of the Pasteur parameter and the incident power flux density, for a nanosphere of radius 50 nm at wavelength 470 nm. (d) The temperature rise (blue curve) and the TCD (red curves) as a function of the power flux density. When T_{rise} is below a specified limit (indicated by the blue circle), the TCD can exceed a certain threshold by adjusting the Pasteur parameter.

Figure S5 shows the temperature analysis for a nanosphere covered by a chiral shell of thickness 10 nm (Figure 1a of the main text). The nanosphere is made of crystalline silicon with realistic permittivity.[5] The permittivity of the chiral sphere is $\varepsilon_r = 1.33^2 - 0.001i$. The thermal conductivity of the surrounding medium is $K_0 = 0.026\ W/m\ K$. The system is illuminated by right- and left-circularly polarized plane waves of the same amplitude, resulting a temperature rise of T_R and T_L for the resonator-shell, respectively. As T_R is very close to T_L, we can define the temperature rise T_{rise} as $(T_R + T_L)/2$. We calculate T_{rise} as a function of the wavelength and the radius of nanosphere, considering incident power flux density of $S_{inc} = 50\ MW\ /\ m^2$ and Pasteur parameter $\kappa = (1-0.01i)\times10^{-2}$ for the chiral shell (Figure S5a).

We see the hottest temperature (indicated by a red circle) occurs at the same point where we get the maximum TCD (i.e., radius $r_i = 50nm$, and $\lambda = 470nm$; see Figure 1 of the main text). We now fix the values for the radius of the nanosphere and the wavelength of the incident light and investigate T_{rise} (Figures

S5b) and TCD (Figures S5c) as a function of the real part of the Pasteur parameter and the incident power flux. The imaginary part of the Pasteur parameter is assumed two orders of magnitude smaller than its real part. We see that T_{rise} is linearly dependent to S_{inc} and being independent to κ, while TCD is linearly scales with both S_{inc} and κ. It is therefore possible to keep S_{inc} below a certain limit to avoid overheating of the chiral samples, and at the same time conduct the experiments with higher Pasteur parameter to go above the sensitivity level of the differential thermal measurements. This has been sketched in Figure S5d, where the left- and the right-axis represent TCD and T_{rise} as a function of incident power flux density S_{inc}. Both TCD and temperature increase linearly by S_{inc}, however at a given temperature (indicated by a blue circle) we can change the TCD by choosing the Pasteur parameter.

Next, we investigate the temperature rise (Figure S6a) and the TCD (Figure S6b) for a 1D array as a function of the number of resonator-shells (*N*). The parameters of the spheres and their covering shells remain consistent with those of the individual resonator discussed above (also the same as Figure 4b of the main text). The spacing between nanoparticles is $d = 8.6r_i$ (i.e., the first order lattice resonance).

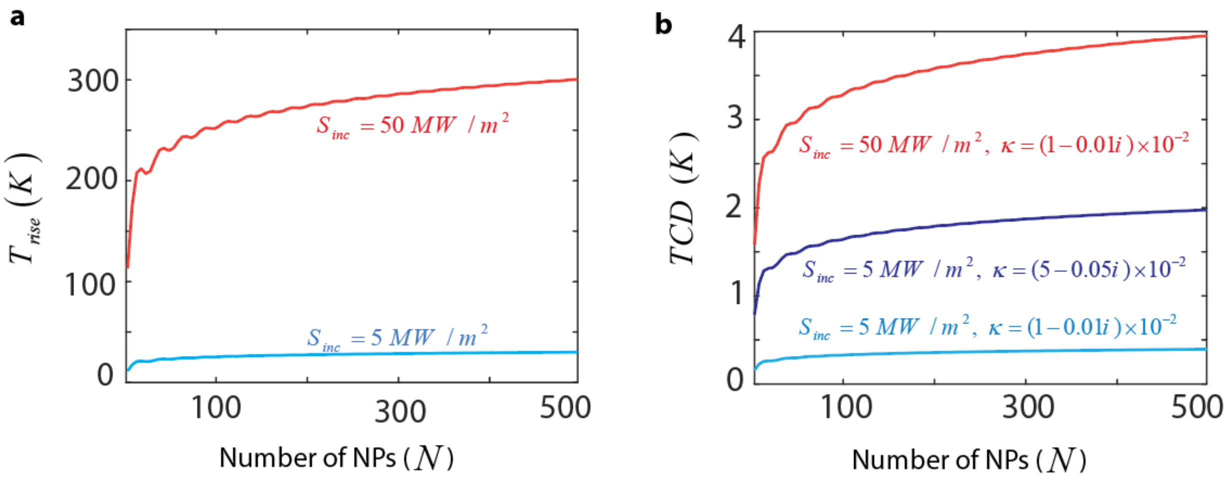

Figure S6. The temperature analysis for a 1D array as a function of the number of resonator-shells (*N*). (a) The temperature is independent of the Pasteur parameter and can be adjusted by changing the incident power flux density. (b) The thermal circular dichroism is dependent on both the power flux density and the Pasteur parameter.

We see that the collective effects in an array made of 500 sphere-shells give rise to 5- and 4-fold enhancements in T_{rise} and TCD (red curves in Figures 6Sa and 6Sb, respectively) compared to an individual resonator-shell (Figure S5a). The high temperature can be reduced linearly by decreasing the incident power flux, as shown by the blue curve in Figure S6a. Reducing the incident power flux density by a factor of 10

results in a proportional decrease in the TCD (light blue curve in Figure S6b). However, the Pasteur parameter can help raise the TCD above a certain threshold (dark blue curve in Figure S6b).

Supporting Section S5. Chirality transfer in an array of nanoresonators covered by chiral shells

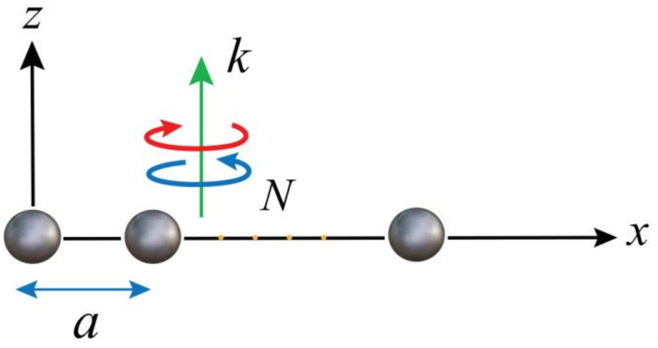

Figure S7. A one-dimensional array of *N* nanospheres arranged along the *x*-axis with spacing *a* and subjected to right- and left-circularly polarized light propagating along the *z*-axis.

Figure S7 depicts a one-dimensional array consisting of *N* nanospheres arranged along the *x*-axis with spacing *a*. The array is illuminated by right- and left-circularly polarized plane waves, for which the electric and the magnetic fields can be expressed as:

$$\begin{aligned} \boldsymbol{E}_{R/L} &= E_0 \exp\left(-ik_0 z\right)\left(\hat{x} \mp i\hat{y}\right) \\ \boldsymbol{H}_{R/L} &= \frac{\pm iE_0}{\eta_0} \exp\left(-ik_0 z\right)\left(\hat{x} \mp i\hat{y}\right) \end{aligned} \qquad \text{(S21)}$$

where the upper (lower) sign in "$\pm$ / $\mp$" is used for the right (left)-circularly polarized excitation, and η_0 is impedance of free space. We model the optical response of this array through Coupled Dipole Approximation (CDA) method, where each nanoparticle is replaced by a pair of electric ($\boldsymbol{p}$) and magnetic ($\boldsymbol{m}$) dipole moments as:

$$\begin{aligned} \boldsymbol{p}_{R/L}^{w.o}\left(\boldsymbol{r}_{0n}\right) &= \alpha_e\left(\boldsymbol{r}_{0n}\right)\boldsymbol{E}_{loc,R/L}^{w.o}\left(\boldsymbol{r}_{0n}\right) \\ \boldsymbol{m}_{R/L}^{w.o}\left(\boldsymbol{r}_{0n}\right) &= \alpha_m\left(\boldsymbol{r}_{0n}\right)\boldsymbol{H}_{loc,R/L}^{w.o}\left(\boldsymbol{r}_{0n}\right) \end{aligned} \qquad \text{(S22)}$$

in which $\boldsymbol{r}_{0n} = d\left(n-1\right)\hat{x}$, $n \in \left(1,2,..,N\right)$ points to the position of the *n-th* nanosphere. The superscript "*w.o*" indicates that there is no chiral shell on the nanospheres yet. The electric and the magnetic dipole

polarizabilities of the nanoparticle are $\alpha_e = \left(-6\pi i\varepsilon_0/k_0^3\right)a_1$ and $\alpha_m = \left(-6\pi i/k_0^3\right)b_1$, where a_1 and b_1 represent the electric and the magnetic dipolar coefficients in the Mie expansion.[6] $\boldsymbol{E}_{loc}\left(\boldsymbol{r}_{0n}\right)$ and $\boldsymbol{H}_{loc}\left(\boldsymbol{r}_{0n}\right)$ are the local electric and magnetic fields at the location of *n-th* nanoparticle, respectively. Such local fields are sum of the incident (Equation S21) and the scattered fields produced by all other nanoparticles in the array and can be expressed as:

$$\begin{aligned}&\boldsymbol{E}_{loc,R/L}^{w.o}\left(\boldsymbol{r}_{0n}\right) = \boldsymbol{E}_{inc,R/L}\left(\boldsymbol{r}_{0n}\right) + \\ &\sum_{i\neq n}\alpha_e\left(\boldsymbol{r}_{0i}\right)\overleftrightarrow{\boldsymbol{G}}_{EP}\left(\boldsymbol{r}_{0n},\boldsymbol{r}_{0i}\right)\cdot\boldsymbol{E}_{loc,R/L}^{w.o}\left(\boldsymbol{r}_{0i}\right) + \alpha_m\left(\boldsymbol{r}_{0i}\right)\overleftrightarrow{\boldsymbol{G}}_{EM}\left(\boldsymbol{r}_{0n},\boldsymbol{r}_{0i}\right)\cdot\boldsymbol{H}_{loc,R/L}^{w.o}\left(\boldsymbol{r}_{0i}\right)\end{aligned} \tag{S23}$$

$$\begin{aligned}&\boldsymbol{H}_{loc,R/L}^{w.o}\left(\boldsymbol{r}_{0n}\right) = \boldsymbol{H}_{inc,R/L}\left(\boldsymbol{r}_{0n}\right) + \\ &\sum_{i\neq n}\alpha_e\left(\boldsymbol{r}_{0i}\right)\overleftrightarrow{\boldsymbol{G}}_{HP}\left(\boldsymbol{r}_{0n},\boldsymbol{r}_{0i}\right)\cdot\boldsymbol{E}_{loc,R/L}^{w.o}\left(\boldsymbol{r}_{0i}\right) + \alpha_m\left(\boldsymbol{r}_{0i}\right)\overleftrightarrow{\boldsymbol{G}}_{HM}\left(\boldsymbol{r}_{0n},\boldsymbol{r}_{0i}\right)\cdot\boldsymbol{H}_{loc,R/L}^{w.o}\left(\boldsymbol{r}_{0i}\right)\end{aligned} \tag{S24}$$

where $\overleftrightarrow{\boldsymbol{G}}_{EP}$ and $\overleftrightarrow{\boldsymbol{G}}_{HP}$ ($\overleftrightarrow{\boldsymbol{G}}_{EM}$ and $\overleftrightarrow{\boldsymbol{G}}_{HM}$) are the electric and the magnetic dyadic Green's functions for radiation of an electric (a magnetic) dipole source in free-space, being expressed as:

$$\overleftrightarrow{\boldsymbol{G}}_{EP}(\boldsymbol{r},\boldsymbol{r}_0) = \frac{k_0^3}{\varepsilon_0}\frac{\exp(-ik_0R)}{4\pi k_0R}\left\{\left(1+\frac{1}{ik_0R}-\frac{1}{k_0^2R^2}\right)\overleftrightarrow{I} - \left(1+\frac{3}{ik_0R}-\frac{3}{k_0^2R^2}\right)\hat{R}\hat{R}\right\} \tag{S25}$$

$$\overleftrightarrow{\boldsymbol{G}}_{HP}(\boldsymbol{r},\boldsymbol{r}_0) = c_0k_0^3\frac{\exp(-ik_0R)}{4\pi k_0R}\left(1+\frac{1}{ik_0R}\right)\hat{R}\times\overleftrightarrow{I} \tag{S26}$$

$$\overleftrightarrow{\boldsymbol{G}}_{EM}(\boldsymbol{r},\boldsymbol{r}_0) = -\eta_0k_0^3\frac{\exp(-ik_0R)}{4\pi k_0R}\left(1+\frac{1}{ik_0R}\right)\hat{R}\times\overleftrightarrow{I} \tag{S27}$$

$$\overleftrightarrow{\boldsymbol{G}}_{HM}(\boldsymbol{r},\boldsymbol{r}_0) = k_0^3\frac{\exp(-ik_0R)}{4\pi k_0R}\left\{\left(1+\frac{1}{ik_0R}-\frac{1}{k_0^2R^2}\right)\overleftrightarrow{I} - \left(1+\frac{3}{ik_0R}-\frac{3}{k_0^2R^2}\right)\hat{R}\hat{R}\right\} \tag{S28}$$

where $\boldsymbol{r}$ and $\boldsymbol{r}_0$ vectors refer to the observation point and the dipole location, respectively. $R = |\boldsymbol{r} - \boldsymbol{r}_0|$ denotes the distance between the dipole source and the observation point, $\hat{R} = \left(\boldsymbol{r} - \boldsymbol{r}_0\right)/R$ is the unit vector pointing from the dipole towards the observation point, and $\overleftrightarrow{I}$ is the unit dyad.[7] Putting all r_{0n} values in Equations S23 and S24, results a self-consistent system of $6N$ equations for each right- or left-circular polarization (i.e., *x*, *y*, and *z* components of $\boldsymbol{E}_{loc}$ and $\boldsymbol{H}_{loc}$ at N locations). Then, arranging this $6N$ equations in a matrix form results:

$$\begin{bmatrix} \boldsymbol{E}^{w.o}_{loc,R/L} \\ \boldsymbol{H}^{w.o}_{loc,R/L} \end{bmatrix}_{6N\times1} = \left[M\right]_{6N\times6N} \begin{bmatrix} \boldsymbol{E}_{inc,R/L} \\ \boldsymbol{H}_{inc,R/L} \end{bmatrix}_{6N\times1} \quad \text{(S29)}$$

where M is the coefficient matrix including Green's functions and polarizabilities. Next, by solving this system of equations for right- and left-circular polarizations separately, we can find the local fields (and subsequently the local induced dipole moments $\boldsymbol{p}^{w.o}_{R/L}$ and $\boldsymbol{m}^{w.o}_{R/L}$) in each case. Once the induced dipole moments are known, we can find the near field around a specific NP (e.g., *k-th* NP) as:

$$\boldsymbol{E}^{w.o}_{R/L}(\boldsymbol{r}_k) = \boldsymbol{E}_{inc,R/L}(\boldsymbol{r}_k) + \sum_{n=1}^{N}\left[\overleftrightarrow{\boldsymbol{G}}_{EP}(\boldsymbol{r}_k,\boldsymbol{r}_{0n})\cdot\boldsymbol{p}^{w.o}_{R/L}(\boldsymbol{r}_{0n}) + \overleftrightarrow{\boldsymbol{G}}_{EM}(\boldsymbol{r}_k,\boldsymbol{r}_{0n})\cdot\boldsymbol{m}^{w.o}_{R/L}(\boldsymbol{r}_{0n})\right] \quad \text{(S30)}$$

$$\boldsymbol{H}^{w.o}_{R/L}(\boldsymbol{r}_k) = \boldsymbol{H}_{inc,R/L}(\boldsymbol{r}_k) + \sum_{n=1}^{N}\left[\overleftrightarrow{\boldsymbol{G}}_{HP}(\boldsymbol{r}_k,\boldsymbol{r}_{0n})\cdot\boldsymbol{p}^{w.o}_{R/L}(\boldsymbol{r}_{0n}) + \overleftrightarrow{\boldsymbol{G}}_{HM}(\boldsymbol{r}_k,\boldsymbol{r}_{0n})\cdot\boldsymbol{m}^{w.o}_{R/L}(\boldsymbol{r}_{0n})\right] \quad \text{(S31)}$$

$\boldsymbol{r}_k = \boldsymbol{r}_{0k} + r_k\,\hat{r}$ refers to the observation point on the surface of *k-th* nanosphere, where $\boldsymbol{r}_{0k} = d\left(k-1\right)\hat{x}$ and r_k are the position and the radius of *k-th* nanosphere, respectively.

Now, we put the chiral shells around the NPs, and assume that the presence of the shell doesn't perturb the near field calculated for the array without the chiral shells (Equations S30 and S31). This assumption is correct as far as the chiral shells are thin and their refractive index is close to the surrounding medium. In this case, the near fields in Equations S30 and S31 induce the electric and magnetic polarization densities inside the chiral shell surrounding *k-th* nanosphere as:

$$\boldsymbol{P}_{R/L}(\boldsymbol{r}_k) = \varepsilon_0\left(\varepsilon_r - 1\right)\boldsymbol{E}^{w.o}_{R/L}(\boldsymbol{r}_k) - i\frac{\kappa}{c_0}\boldsymbol{H}^{w.o}_{R/L}(\boldsymbol{r}_k) \quad \text{(S32)}$$

$$\boldsymbol{M}_{R/L}(\boldsymbol{r}_k) = i\frac{\kappa}{\eta_0}\boldsymbol{E}^{w.o}_{R/L}(\boldsymbol{r}_k) \quad \text{(S33)}$$

where, we have used the constitutive relations $\boldsymbol{D} = \varepsilon\boldsymbol{E} - i\frac{\kappa}{c_0}\boldsymbol{H} = \varepsilon_0\boldsymbol{E} + \boldsymbol{P}$ and

$\boldsymbol{B} = \mu\boldsymbol{H} + i\frac{\kappa}{c_0}\boldsymbol{E} = \mu_0\left(\boldsymbol{H} + \boldsymbol{M}\right)$ describing the chiral media. To calculate the chirality transfer,[7] we need to find the back-action of such polarization (Equations S32 and S33) on the *k-th* nanoresonator (located at $\boldsymbol{r}_{0k}$). Such back coupling alters the local fields at the position of resonator being otherwise equal for right-

and left-circular polarizations (i.e., $\left|\boldsymbol{E}_{loc,R}^{w.o}\left(\boldsymbol{r}_{0k}\right)\right|=\left|\boldsymbol{E}_{loc,L}^{w.o}\left(\boldsymbol{r}_{0k}\right)\right|$, $\left|\boldsymbol{H}_{loc,R}^{w.o}\left(\boldsymbol{r}_{0k}\right)\right|=\left|\boldsymbol{H}_{loc,L}^{w.o}\left(\boldsymbol{r}_{0k}\right)\right|$). We write updated local fields as:

$$\begin{aligned}&\boldsymbol{E}_{loc,R/L}^{w}\left(\boldsymbol{r}_{0k}\right)=\boldsymbol{E}_{loc,R/L}^{w.o}\left(\boldsymbol{r}_{0k}\right)+\\&\int_{shell}\overleftrightarrow{\boldsymbol{G}}_{EP}(\boldsymbol{r}_{0k},\boldsymbol{r}_k)\cdot\left[\varepsilon_0\left(\varepsilon_r-1\right)\boldsymbol{E}_{R/L}^{w.o}\left(\boldsymbol{r}_k\right)-i\frac{\kappa}{c_0}\boldsymbol{H}_{R/L}^{w.o}\left(\boldsymbol{r}_k\right)\right]dv+\int_{shell}\overleftrightarrow{\boldsymbol{G}}_{EM}(\boldsymbol{r}_{0k},\boldsymbol{r}_k)\cdot\left[i\frac{\kappa}{\eta_0}\boldsymbol{E}_{R/L}^{w.o}\left(\boldsymbol{r}_k\right)\right]dv\end{aligned}\qquad\text{(S34)}$$

$$\begin{aligned}&\boldsymbol{H}_{loc,R/L}^{w}\left(\boldsymbol{r}_{0k}\right)=\boldsymbol{H}_{loc,R/L}^{w.o}\left(\boldsymbol{r}_{0k}\right)+\\&\int_{shell}\overleftrightarrow{\boldsymbol{G}}_{HP}(\boldsymbol{r}_{0k},\boldsymbol{r}_k)\cdot\left[\varepsilon_0\left(\varepsilon_r-1\right)\boldsymbol{E}_{R/L}^{w.o}\left(\boldsymbol{r}_k\right)-i\frac{\kappa}{c_0}\boldsymbol{H}_{R/L}^{w.o}\left(\boldsymbol{r}_k\right)\right]dv+\int_{shell}\overleftrightarrow{\boldsymbol{G}}_{HM}(\boldsymbol{r}_{0k},\boldsymbol{r}_k)\cdot\left[i\frac{\kappa}{\eta_0}\boldsymbol{E}_{R/L}^{w.o}\left(\boldsymbol{r}_k\right)\right]dv\end{aligned}\qquad\text{(S35)}$$

where the superscript "*w*" refers to the presence of the chiral shell around nanospheres in the array. The dipole moments induced in the *k-th* nanoresonator in the presence of the chiral shells are obtained as $\boldsymbol{p}_{R/L}^{w}\left(\boldsymbol{r}_{0k}\right)=\alpha_e\left(\boldsymbol{r}_{0k}\right)\boldsymbol{E}_{loc,R/L}^{w}\left(\boldsymbol{r}_{0k}\right)$ and $\boldsymbol{m}_{R/L}^{w}\left(\boldsymbol{r}_{0k}\right)=\alpha_m\left(\boldsymbol{r}_{0k}\right)\boldsymbol{H}_{loc,R/L}^{w}\left(\boldsymbol{r}_{0k}\right)$, where now $\left|\boldsymbol{p}_{R}^{w}\left(\boldsymbol{r}_{0k}\right)\right|\neq\left|\boldsymbol{p}_{L}^{w}\left(\boldsymbol{r}_{0k}\right)\right|$ and $\left|\boldsymbol{m}_{R}^{w}\left(\boldsymbol{r}_{0k}\right)\right|\neq\left|\boldsymbol{m}_{L}^{w}\left(\boldsymbol{r}_{0k}\right)\right|$ due to the chirality transfer. Once the updated dipole moments are determined, we can obtain the scattered and the extinct powers by the *k-th* NP and corresponding to the right- and left-circularly polarized excitations as:

$$P_{R/L}^{sca,k}=\frac{\eta_0 k_0^4}{12\pi}\left(c_0^2\left|\boldsymbol{p}_{R/L}^{w}\left(\boldsymbol{r}_{0k}\right)\right|^2+\left|\boldsymbol{m}_{R/L}^{w}\left(\boldsymbol{r}_{0k}\right)\right|^2\right)\qquad\text{(S36)}$$

$$P_{R/L}^{ext,k}=\frac{\omega_0}{2}\operatorname{Im}\left[\boldsymbol{E}_{loc,R/L}^{w.o}\left(\boldsymbol{r}_{0k}\right)\cdot\boldsymbol{p}_{R/L}^{*w}\left(\boldsymbol{r}_{0k}\right)+\mu_0\boldsymbol{H}_{loc,R/L}^{w.o}\left(\boldsymbol{r}_{0k}\right)\cdot\boldsymbol{m}_{R/L}^{*w}\left(\boldsymbol{r}_{0k}\right)\right]\qquad\text{(S37)}$$

Finally, we can find the differential scattered, extinct, and absorbed powers by the *k-th* NP as:

$$\Delta P^{sca,k}=P_R^{sca,k}-P_L^{sca,k}\qquad\text{(S38)}$$

$$\Delta P^{ext,k}=P_R^{ext,k}-P_L^{ext,k}\qquad\text{(S39)}$$

$$\Delta P^{abs,k}=\Delta P^{ext,k}-\Delta P^{sca,k}\qquad\text{(S40)}$$

Supporting Section S6. Comparison between the chiral sensitivity of CD and TCD

We consider two systems, as shown in Figure S8: (1) a chiral slab with thickness l and Pasteur parameter κ (Figure S8a). This system is intended for Circular Dichroism (CD) studies. (2) A 2D array of chiral

spheres, composed of N spheres, each with radius r and separated by a distance $d = 3r$ (Figure S8b). There are $\sqrt{N}$ spheres along each direction. This system is considered for Thermal Circular Dichroism (TCD) studies. Both systems are illuminated by right- and left-circularly polarized light of the equal amplitudes E_0 (Equation S21). We selected these two systems because we have analytical formulas for the CD of the first system and for the TCD of the second. In our comparison, we assume the diameter of the chiral spheres in the array to be equal to the thickness of the chiral slab, both set at 10 nm ($l = 2r = 10nm$). It is important to note that the amount of chiral material in system A is greater than in system B, as the chiral slab is continuous, whereas the array is finite and contains empty space between the spheres. Our goal is to calculate the CD of system A and compare it to the TCD of system B, demonstrating that TCD can detect lower molecular concentrations, thereby offering better sensitivity.

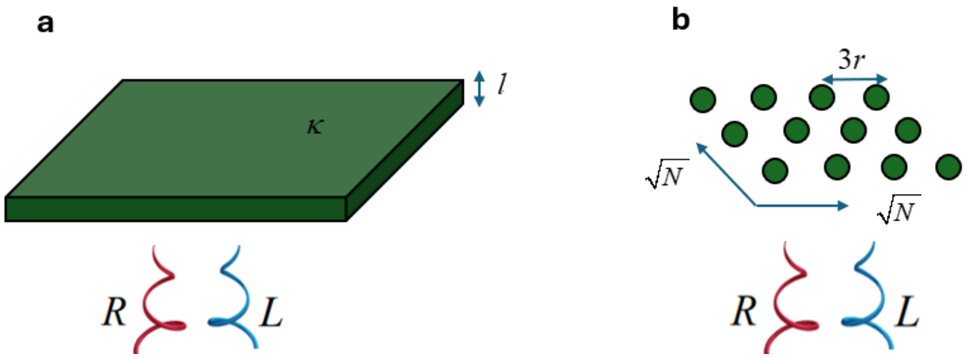

Figure S8. (a) A chiral slab of thickness l and Pasteur parameter κ for CD studies. (b) An array made of $\sqrt{N} \times \sqrt{N}$ chiral spheres for TCD studies. The spheres have a diameter $2r$ and a spacing $3r$ (center-to-center) between them.

As derived in section Equation S10, the imaginary part of Pasteur parameter, $\mathrm{Im}(\kappa)$, is linearly proportional to the concentration C. Without loss of generality, we consider $\mathrm{Im}(\kappa) = 10^{-6}$ at optical wavelengths.[2] Substituting $l = 10nm$ and $\mathrm{Im}(\kappa) = 10^{-6}$ in Equation S4, we obtain $CD \approx 0.008$ mdeg at wavelength 470 nm. Even with high CD sensitivities, such as 1 mdeg, this value remains more than two orders of magnitude below the detection limit ($\frac{1 \text{ mdeg}}{0.008 \text{ mdeg}} = 125$).

Next, we quantify the TCD for system B. The TCD for a single chiral sphere is given by Equation S19.

Substituting the $\lambda_0 = 470nm$, $r = 5nm$, $\left(E_0^2 / \eta_0\right) = 5\times10^7 W / m^2$, $\mathrm{Im}(\kappa) = 10^{-6}$, $K_0 = 0.026\ W / m\ K$, and $\varepsilon_r = 1.33^2 - 0.001i$, we obtain $TCD \approx 6.8\times10^{-4} mK$. Now, assuming $N \approx 10^6$ (1000 chiral spheres in each direction) and distancing $d \approx 3r = 15nm$ (array size $15\mu m$), the array thermal factor (ATF) is obtained as 1150 which is plotted in Figure S9. Such ATF results $TCD \approx 0.8mK$ for the central nanosphere in the array.

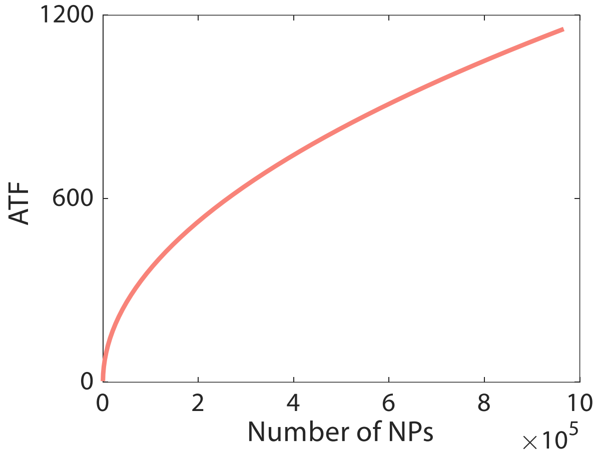

Figure S9. The array thermal factor (ATF; see Equations 3,4 in the main manuscript), for a 2D array of chiral spheres each with radius $r = 5nm$ and spacing $3r = 15nm$ between them.

Millikelvin accuracy in temperature measurement has already been demonstrated using advanced nanothermometry techniques such as probing of single-atomic defects in diamond.[8-9] Moreover, there are variety of techniques such as optical interferometry and thermoreflectance being able to address the required temperature measurement resolutions along with nanometric spatial resolutions. [10]

References

(1) Fasman, G. D. Circular Dichroism and the Conformational Analysis of Biomolecules; Springer Science & Business Media, 2013.
(2) Garcia-Guirado, Jose, et al. "Enhanced chiral sensing with dielectric nanoresonators." Nano letters 20.1 (2019): 585-591.
(3) Mohammadi, Ershad, T. V. Raziman, and Alberto G. Curto. "Nanophotonic chirality transfer to dielectric Mie resonators." Nano Letters 23.9 (2023): 3978-3984.
(4) Lindell, I. V; Sihvola, A. H. Quasi-Static Analysis of Scattering from a Chiral Sphere. J. Electromagn. Waves Appl. 1990, 4 (12), 1223–1231.

(5) Aspnes, D. E.; Studna, A. A. Dielectric Functions and Optical Parameters of Si, Ge, GaP, GaAs, GaSb, InP, InAs, and InSb from 1.5 to 6.0 eV. Phys. Rev. B 1983, 27 (2), 985.
(6) Bohren, Craig F., and Donald R. Huffman. Absorption and scattering of light by small particles. John Wiley & Sons, 2008.
(7) Jackson, J. D. Classical Electrodynamics; Wiley, 1999.
(8) Kucsko, Georg, et al. "Nanometre-scale thermometry in a living cell." *Nature* 500.7460 (2013): 54-58.
(9) Neumann, Philipp, et al. "High-precision nanoscale temperature sensing using single defects in diamond." *Nano letters* 13.6 (2013): 2738-2742.
(10) Brites, Carlos DS, et al. "Thermometry at the nanoscale." *Nanoscale* 4.16 (2012): 4799-4829
